# Transcriptional silencing of a conjugative Sinorhizobium megaplasmid upon transfer to different rhizobial species

**DOI:** 10.3389/fmicb.2026.1977405

**Published:** 2026-10-22

**Authors:** Cristina de la Cruz, Laura Cervantes, Gonzalo Torres-Tejerizo, David Romero

**Affiliations:** 1 Programa de Ingeniería Genómica, Centro de Ciencias Genómicas, Universidad Nacional Autónoma de México, Cuernavaca, Mexico; 2 Departamento de Ciencias Biológicas, Facultad de Ciencias Exactas, Instituto de Biotecnología y Biología Molecular (IBBM)—CCT CONICET—La Plata, Universidad Nacional de la Plata, La Plata, Argentina

**Keywords:** gene silencing, horizontal transfer, megaplasmid, plasmid transfer, transcriptome regulation

## Abstract

Horizontal gene transfer of large conjugative megaplasmids drives bacterial evolution, however, it forces new hosts to rapidly adapt to a massive influx of exogenous DNA; even the acquisition of small plasmids can have variable effects on host genome activity and fitness. In *Sinorhizobium fredii* GR64 (symbiont of the common bean) the megaplasmid pSfr64a (191 kb) displays a high conjugative transfer rate. Interestingly, when pSfr64a is transferred to *Rhizobium etli* (another bean symbiont) conjugative transfer from this host became nearly undetectable, whereas in *Agrobacterium tumefaciens* it is maintained at a high rate. To unravel the host-plasmid dynamics driving this host-specific response, we undertook a transcriptome analysis of pSfr64a in different hosts. The results reveal that in its original host, pSfr64a transcriptional activity is centered in four zones, including the conjugative zone. Expression of these zones is markedly quelled when pSfr64a resides in *R. etli*, explaining the reduction in conjugative activity. Crucially, this silencing ensures minimal effects on the host’s transcriptional activity, allowing *R. etli* to adapt to the megaplasmid with zero fitness cost. In contrast, a weak silencing in *A. tumefaciens* allows conjugation, but triggers a widespread transcriptional reorganization across both linear and circular chromosomes, severely penalizing host fitness. Although inactivation of the xenogeneic silencer MucR in *R. etli* leads to a partial recovery of plasmid expression, it does not restore conjugation. These results highlight a fundamental evolutionary conflict: the successful adaptation to a newly acquired megaplasmid depends on a hierarchical, host-specific silencing architecture that must tame the biological cost of the plasmid’s “selfish mode.”

## 1 Introduction

Plasmid transfer by conjugation is a quotidian endeavor in the bacterial world. Plasmids are a reservoir of useful genes that aid in niche expansion and adaptation (Norman et al., 2009), but also a potent drive for bacterial evolution. A central topic is understanding the mechanism of conjugation and their variations (Smillie et al., 2010; Thomas and Nielsen, 2005). Conjugation of small (<50 kb) and medium-sized plasmids (50–100 kb) aid in the acquisition of characteristics as diverse as antibiotic resistance, xenobiotic degradation and virulence towards plants and animals. There is also a growing recognition that plasmid acquisition may entail a significant metabolic burden to the recipient cell, requiring specific countermeasures to limit the cost (Curtsinger et al., 2024; San Millan and MacLean, 2017; San Millan et al., 2015; Stalder et al., 2017; Valle et al., 2021; Cyriaque et al., 2024). These countermeasures imply a strict regulation of conjugative activity, but also the action of global silencers that aid in reducing the cost, as well as metabolic and genetic adaptations (Curtsinger et al., 2024).

This demand is even higher for bacterial groups that harbor divided genomes, characterized by the presence of one or two chromosomes plus several large plasmids (100 kb–2 Mb), commonly referred as megaplasmids (diCenzo and Finan, 2017; Hall et al., 2022). Several of these megaplasmids may be conjugative, and it is reasonable to expect that acquisition of such large genomic elements would impact significantly bacterial fitness. Research has concentrated on the characteristics encoded in megaplasmids, as well as how they regulate its conjugative ability. A common mechanism for nitrogen-fixing Rhizobium bacteria, as well as in the plant pathogen Agrobacterium, is a positive regulation of conjugation by quorum sensing. This mechanism senses cell density by detecting the concentration of autoinducer acyl-homoserine lactones (AHLs), which in turn interact with the positive regulator TraR, activating the transcription of operons participating in conjugation (the Dtr and Mpf systems, after DNA transfer and mating pair formation respectively). For Rhizobium and Agrobacterium, AHLs-based regulatory systems are common, although there are examples of negative regulatory systems for conjugation (Bañuelos-Vazquez et al., 2017; Ding and Hynes, 2009; Pérez-Mendoza et al., 2005; Romero and Brom, 2004). Evaluations of the burden of megaplasmids to the host cell have lagged those for small and medium-sized plasmids, although there are studies that reveal significant costs upon acquisition of megaplasmids from *Agrobacterium tumefaciens* (Platt et al., 2012), *Pseudomonas syringae* (Baltrus et al., 2021; Romanchuk et al., 2014; Smith et al., 2022) and *Sinorhizobium meliloti* (Finan et al., 1986).

This study was motivated by the unusual characteristics of pSfr64a, a self-conjugative plasmid (191 kb) from *Sinorhizobium fredii* GR64 (Herrera-Cervera et al., 1999). Sequence analysis of pSfr64a reveal a mosaic composition, where the conjugation region is highly similar to the conjugative region of pRet42a from *Rhizobium etli* CFN42, another sector to part of the pSym of *R. etli* CFN42 and the reminder to sectors from the Sinorhizobium chromosome (Cervantes et al., 2011). pSfr64a can be transferred at a high frequency from its native background to a variety of hosts, including *R. etli* and *A. tumefaciens*, and its conjugative ability is regulated by quorum sensing. Interestingly, when pSfr64a is in a *R. etli* background, transfer frequency is severely reduced, but when is in an *A. tumefaciens* background, high transfer frequency is maintained. Reduced transfer frequency in *R. etli* cannot be improved by addition of transcriptional regulators that conceivably act on the Dtr or Mpf systems (Cervantes et al., 2019).

Under the idea that these differences in conjugative ability may be due to global silencing of pSfr64a acting in a host-specific way, we undertook a comparative transcriptomic analysis of the pSfr64a in these different species. These studies were extended to include the effect of pSfr64a on host genome expression, as well as evaluations of its effects on fitness. Results indicate that there is host-specific silencing of the major transcriptional units of pSfr64a, including the conjugative region. When the silencing is not efficient, it may have a major effect on fitness.

## 2 Materials and methods

### 2.1 Bacterial strains, growth conditions and plasmids used

Bacterial strains and plasmids employed in this work are listed in Supplementary Table S1. *R. etli* and *S. fredii* strains were grown at 30 °C in Peptone-Yeast extract (PY) rich medium supplemented with 7 mM calcium chloride (Noel et al., 1984). *A. tumefaciens* and *E. coli* strains were grown in LB rich medium at 30 °C (*A. tumefaciens*) or 37 °C (*E. coli*). For growth in defined minimal medium, MMY minimal medium with sodium succinate and ammonium chloride (10 mM each) was used, supplemented with biotin at 1 mg l^−1^ (Bravo and Mora, 1988). Antibiotics were used at the following concentrations (in μg ml^−1^): nalidixic acid, 20; gentamicin, 30; neomycin, 60; rifampicin, 50 and spectinomycin, 75.

### 2.2 Growth measurements

All growth experiments started by streaking the desired strains from frozen glycerol stocks onto the appropriate solid medium and incubated by 72 h. The streaked strains were used to inoculate 5 mL cultures, grown overnight. For quantitation of GFP fluorescence, cultures were set up in 96-well assay plates at an initial A_600_ of 0.01 and incubated at 30 °C in a Synergy HT reader (Biotek). Readings for cell density (A_600_) and GFP cell fluorescence were taken automatically every 20 min, for periods of up to 24 h; fluorescence values were normalized according to cell density, and values were graphed for determination of area under the curve (AUC). At least three biological replicates, each with three technical replicates, were done for every condition and strain.

For determination of growth kinetics, cultures of strains *R. etli* CFN2001, *R. etli* CFN2001/pSfr64a, *A. tumefaciens* GMI9023 and *A. tumefaciens* GMI9023/pSfr64a were set up in 96-well assay plates at an initial A_600_ of 0.1 and incubated at 30 °C in a Synergy HT reader (Biotek), with periodic recordings of A_600_. From seven to nine biological replicates were done for each strain used, with three technical replicates per each biological replicate. Maximum specific growth rate (*μ*_max_, in h^−1^) was evaluated from the growth curves, as the slope obtained during the exponential phase (2–8 h).

### 2.3 Bacterial competence assays

For this study, strains to be competed were grown overnight in PY medium at 30 °C. Once grown, strains were inoculated pairwise at an A_600_ of 0.05 each on 25 mL-PY medium cultures and grown with shaking at 30 °C. Competitions were set up with strains *R. etli* CFN2001 vs. *R. etli* CFN2001/pSfr64a and *A. tumefaciens* GMI9023 vs. *A. tumefaciens* GMI9023/pSfr64a. Evaluations of viable cell number of each strain were done at 0 and 8 h, by dilution and selective plating of mixed cultures, distinguishing strains that harbor pSfr64a by its resistance to spectinomycin. Three biological replicates, each with three technical replicates, were done for every competition. Results are expressed as a Competitive Index (CI), according to the formula CI = (*N_f pSfr64a_*/*N_f_*)/(*N_i pSfr64a_*/*N_i_*), where *N_i_* and *N_f_* are the number of cells of the parental strain lacking pSfr64a at the beginning and the end of the competition, and *N_i pSfr64a_* and *N_f pSfr64a_* correspond to the initial and final number of cells of a transconjugant strain of the same species carrying pSfr64a. To evaluate competitive index at longer times (48 and 72 h), competition cultures were grown for 24 h, diluted in a 1:25 proportion (vol/vol) with fresh medium and grown for an additional 24 h (to reach 48 h); for competitions reaching 72 h, the procedure was repeated with cells from the previous competition.

### 2.4 Bacterial matings

Conjugation experiments were performed as reported previously (Brom et al., 2004). For a standard mating experiment, overnight cultures in rich medium of recipients and donors were mixed in a 2:1 volume ratio (total, 300 μL) and spread onto PY plates. After 24 h of incubation at 30 °C, cells from the mating mixture were scraped off the plates and resuspended in 1 mL of 10 mM MgSO_4_-0.01% (vol/vol) Tween 40. Serial dilutions were plated on appropriate selective media to determine the number of donors, recipients and transconjugants in each mating. Conjugation frequency was expressed as number of transconjugants per output recipient. To verify the transfer of pSfr64a, plasmid content in the transconjugants was analyzed in agarose gels by a modified Eckhardt in-gel lysis technique (Eckhardt, 1978; Hynes and McGregor, 1990).

### 2.5 PCR procedures

Most PCR procedures were done with recombinant Taq DNA polymerase in a Mastercycler 5,330 (Eppendorf), except for construction of the *gfpmut3*ΩSp allele, where the Phusion High Fidelity DNA Polymerase was used (see below). PCR conditions consisted of 35 cycles of denaturation (2 min at 94 °C), annealing (2 min at variable temperatures depending on the primers used, Supplementary Table S2), extension (1 min per kb at 72 °C). All reactions were ended by an incubation of 5 min at 72 °C. Fragments obtained were analyzed by gel electrophoresis in 1% agarose gels.

### 2.6 Introduction of a *gfpmut3*ΩSp allele into a pSfr64a

To introduce a fluorescent marker into pSfr64a (Gene Bank accession number CP137019.1) in a region not affecting conjugation, an intergenic region was chosen between the genes 5 (SFGR64A_32300) and 6 (SFGR64A_32305). The left part of this region was amplified with primers P1 and P2 (having built-in sites for EcoRI and BamHI, respectively, Supplementary Table S2) and the right part with primers P3 and P4 (built-in sites BamHI-NotI and HindIII). Both products were column-purified, digested with BamHI and ligated with T4 DNA ligase (Thermo Scientific). Upon ligation, the desired product was amplified with primers P1 and P4, column-purified, digested with EcoRI and HindIII and ligated into similarly restricted pK18mobSacB (Schäfer et al., 1994) giving rise to pCC1 int. A 4144 bp fragment containing the *gfpmut3*ΩSp marker was obtained by NotI digestion of pGX541 (Torres-Tejerizo et al., 2015), gel-purified and ligated into the NotI site of pCC1 int. The resulting construction (pCC1 int. *gfpmut3*ΩSp) was transformed into *E. coli* S17-1 and transferred by conjugation to *S. fredii* GR64, selecting for Nal^r^ Sp^r^ transconjugants. To select for transconjugants in which allelic exchange had occurred, these were grown in PY medium containing saccharose (12.5% w/v) and verifying that had a Sp^r^ Kan^s^ phenotype. Further confirmation was done by PCR amplification with primer pairs CF1-CasNot-Ter-out (amplicon size, 649 bp) and CF2-pneo-out (amplicon size, 942 bp).

### 2.7 Construction of a *R. etli mucR* mutant

To construct a knockout mutant in the *mucR* gene of *R. etli*, vector-insertion mutagenesis was employed. Briefly, an internal fragment of the *mucR* gene, flanked by built-in HindIII and BamHI restriction sites, was amplified by PCR, digested with restriction enzymes and cloned into the suicide plasmid pk18mob (Schäfer et al., 1994). Ligation mixtures were transformed into cells of *E. coli* DH5α, made competent by a RbCl_2_-CaCl_2_ procedure. The resulting plasmid pK18mob::*mucR* was then transformed into *E. coli* S17-1. To introduce the mutation into *R. etli*, a biparental plate mating using *E. coli* S17-1/pK18mob::*mucR* as a donor and *R. etli* CFN2001/pSfr64a as a recipient was carried out. *R. etli* transconjugants containing insertion of pK18mob::*mucR* were selected onto PY medium containing nalidixic acid and kanamycin. To verify that the *mucR* gene of *R. etli* was interrupted by insertion of the vector, PCR with external primers (Supplementary Table S2) was done.

### 2.8 Quantitative RT-qPCR analysis

To quantify the expression levels for selected genes on pSfr64a, RT-qPCR analyses were done. Primers were designed for each gene (Supplementary Table S2) with Primer3 software,^1^ and its specificity was verified by BLASTN analysis. Extraction of total RNA from *S. fredii* GR64, *R. etli* CFN2001/pSfr64a and *A. tumefaciens* GMI9023/pSfr64a was done from mid-log phase cultures (A_600_ = 0.5) in PY rich medium, using the Trizol reagent (Zymo Research) according to the instructions of the manufacturer. DNA was removed with DNAse I (Thermo Scientific). RNA was quantified in a Nanodrop 200c (Thermo Scientific), and integrity of the RNA was verified by agarose gel electrophoresis for each sample. cDNA was synthesized with the Revert Aid H Minus First Strand cDNA Kit (Thermo Scientific). Determinations by qPCR were executed in a Step One Plus device (Applied Biosystems), using the Maxima SYBR Green/ROX qPCR Kit, Master Mix 2x (Thermo Scientific). Runs were started with an initial denaturation step consisting of 2 min at 50 °C and 10 min at 95 °C, followed by a thermal cycling regime composed of 40 cycles of denaturation at 95 °C for 15 s and annealing/extension at variable temperatures depending on the primer (Supplementary Table S2) for 30 s. Primers for the *rpoA* gene of each strain were used as control. Quantification ratios were calculated based on the cycle threshold (CT) values. Three biological replicates were performed, each with three technical replicates.

### 2.9 RNAseq analyses

Cells from strains *S. fredii* GR64, *R. etli* CFN2001, *R. etli* CFN2001/pSfr64a, *A. tumefaciens* GMI9023 and *A. tumefaciens* GMI9023/pSfr64a were grown to mid-log phase (A_600_ 0.5, 10^8^ cells) in liquid PY rich medium, spun down and stored on RNAlater (Thermo-Fisher). Three biological replicates were set up for each strain analyzed. RNA extraction, rRNA depletion, library preparation and DNBSEQ™ Sequencing was done by BGI (Hong Kong). Subsequent analyses were done in the AKNA server of the Centro de Ciencias Genómicas-UNAM. Raw readings were evaluated with FastQC. Reads were cleaned with Trimmomatic v.0.36 (Bolger et al., 2014). To evaluate the expression of pSfr64a in strains *S. fredii* GR64, *R. etli* CFN2001/pSfr64a and *A. tumefaciens* GMI9023/pSfr64a, paired-end reads were mapped towards the *S. fredii* pSfr64a sequence (Supplementary Table S3) using Bowtie2 aligner v.2.2.6 (Langmead and Salzberg, 2012). Counts per gene were done with HT-seqCounts 2.0 (Putri et al., 2022). Differential expression analysis was done with DESeq2 (Love et al., 2014).

To evaluate the effect of the addition of pSfr64a on genome expression, paired-end reads of *R. etli* CFN2001, *R. etli* CFN2001/pSfr64a, *A. tumefaciens* GMI9023 and *A. tumefaciens* GMI9023/pSfr64a were mapped towards the appropriate genome sequences (Supplementary Table S3) using Bowtie2 aligner v.2.2.6, followed by analyses with HT-seqCounts 2.0 and DESeq2. Essentiality of DEGs in *A. tumefaciens* was assessed from published data (Curtis and Brun, 2014).

### 2.10 Statistical analysis

Data analysis and visualization were performed using R software and Rstudio, with plots generated via ggplot2 package. Given the inherent biological variance of the bacterial population and sample sizes, non-parametric statistical methods were employed for hypothesis testing. The Area Under the Curve (AUC) for fluorescence kinetics was calculated using the growthcurver package (Sprouffske and Wagner, 2016). To determine statistical significance among three groups, a Kruskal-Wallis test was used, followed by pairwise Wilcoxon rank sum tests to identify specific group differences. For RT-qPCR analyses, data are presented as means +/− standard deviation. In all the experiments an alpha level of *p* < 0.05 was considered statistically significant.

## 3 Results

### 3.1 Conjugation efficiency of pSfr64a is modified upon transfer to other rhizobial hosts

To better evaluate the degree of reduction of conjugation efficiency of pSfr64a upon transfer to different rhizobial hosts, a derivative of pSfr64a was constructed, harboring a gene for the green fluorescent protein (GFP) under the control of the strong *nptII* promoter (Materials and Methods). This gene was inserted in the intergenic region (nt 4,704–5,001) separating genes 5 (SFGR64A_32300) and 6 (SFGR64A_32305) on pSfr64a. This region is far from the region harboring the Dtr (*traCDG* and *traAFBH*, nt 168,359–176,754) and Mpf components (*trbCDEJKLFHI*, nt 180,026–189,536).

When this plasmid derivative resides in *S. fredii* GR64, it maintains a high frequency of transfer to different hosts, such as *R. etli* CFN2001 (Table 1, row 1) or towards two different strains of *A. tumefaciens* (Table 1, rows 2 and 3). In contrast, when pSfr64a::*gfp* resides in *R. etli* CFN2001, transfer frequency to *A. tumefaciens* is reduced by seven orders of magnitude (Table 1, row 4). This result is not due to some sort of incompatibility of matings between *R. etli* and *A. tumefaciens*. If the natural conjugative plasmid of *R. etli* (pRet42a::*gfp*) is residing in *R. etli* CFN2001, is readily transferred to *A. tumefaciens* (Table 1, row 6), at the high conjugative frequency characteristic of this plasmid (Tun-Garrido et al., 2003).

**Table 1:** Conjugation efficiency of pSfr64a is suppressed upon transfer to *R. etli*.

Donor	Plasmid transferred	Recipient	Conjugation frequency (×10^−4^ ± SD)^a^
*S. fredii* GR64-3	pSfr64a::*gfp*	*R. etli* CFN2001	3,800 ± 2,800
*S. fredii* GR64-3	pSfr64a::*gfp*	*A. tumefaciens* GMI9023	210 ± 230
*S. fredii* GR64-3	pSfr64a::*gfp*	*A. tumefaciens* UIA143	430 ± 3.9
*R. etli* CFN2001/pSfr64a::*gfp*	pSfr64a::*gfp*	*A. tumefaciens* UIA143	0.0001 ± 0.00008
*A. tumefaciens* GMI9023/pSfr64a::*gfp*	pSfr64a::*gfp*	*A. tumefaciens* UIA143	380 ± 4.1
*R. etli* CFN2001/pRet42a::*gfp*	pRet42a::*gfp*	*A. tumefaciens* UIA143	3 ± 1

^a^Conjugation frequency is expressed as transconjugants per recipient cell. Data are means of three biological replicates ± standard deviation.

Interestingly, when pSfr64a::*gfp* is residing in *A. tumefaciens* and is transferred to another *A. tumefaciens* strain, it displays a high frequency of transfer (Table 1, row 5). This indicates that pSfr64a::*gfp* contains the necessary elements for high-frequency conjugation from *A. tumefaciens*. Strikingly, these elements appear to be insufficient to foster transfer in *R. etli*. A plausible alternative to explain these results would be xenogeneic silencing of the conjugative regions of pSfrGr64a, operating in the *R. etli* background.

### 3.2 Reduced expression of a foreign gene (*gfp*) upon transfer of pSfr64a::*gfp* to *R. etli*

If the reduced conjugation frequency of pSfr64a::*gfp* in *R. etli* is due to xenogeneic silencing, it is reasonable to expect that this silencing may be extended to other plasmid regions. Therefore, expression of the foreign *gfp* gene added to pSfr64a was quantified in the different hosts employed. As shown in Figure 1, high levels of fluorescence were detected both in *S. fredii* and *A. tumefaciens*, but is reduced, at least three-fold, when pSfr64a::*gfp* was transferred to *R. etli* CFN2001. This reduction in fluorescence cannot be attributed to the absence of regulatory elements, since the *gfp* gene is expressed from a strong, constitutive promoter. Moreover, when the same *gfp* gene was introduced in the *R. etli* plasmid pRet42a::*gfp,* high levels of fluorescence were seen (Bañuelos-Vázquez et al., 2019). These results are better explained by invoking a silencing effect, perhaps mediated by xenogeneic silencers.

**Figure 1:**
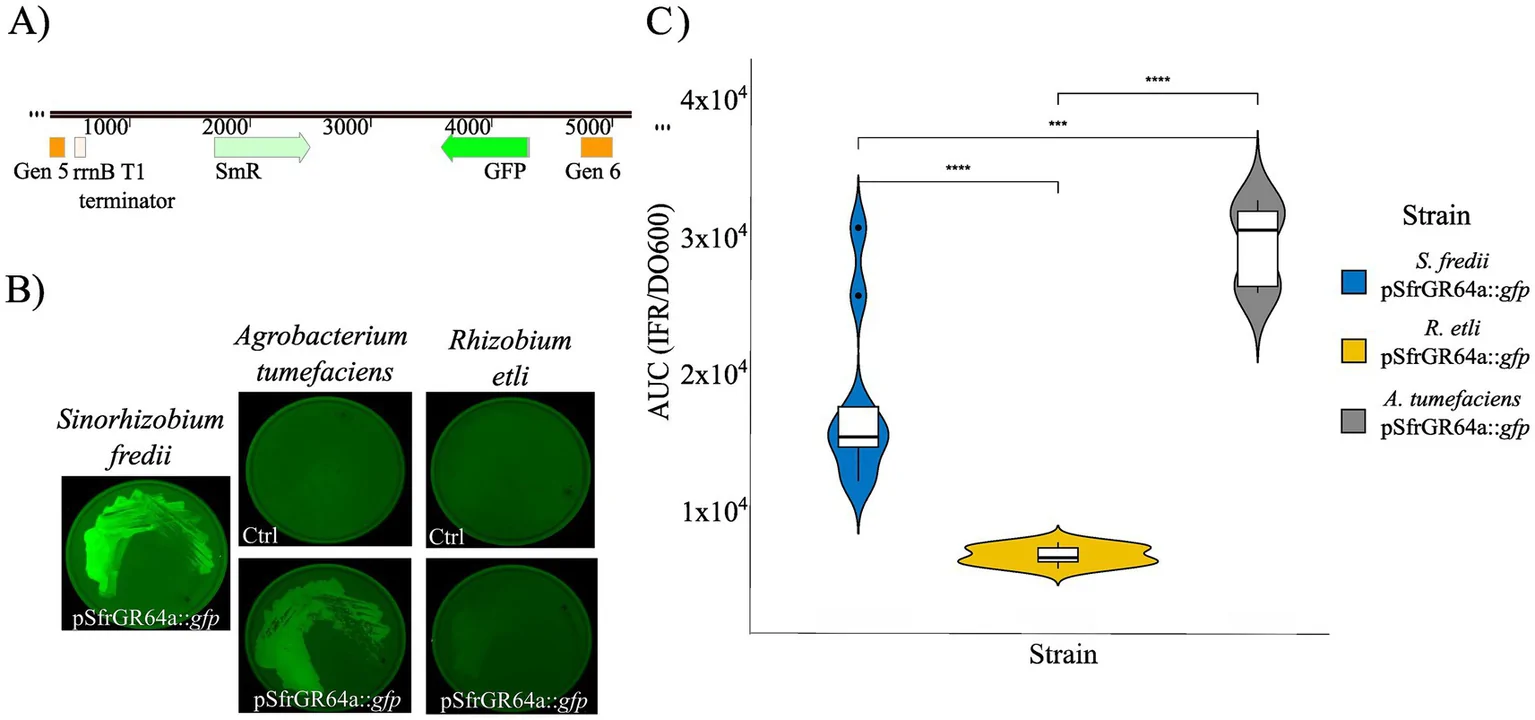
**(A)** Representation of the construction pSfr64a::*gfpmut3.* **(B)** Fluorescence observed in Petri dishes containing the indicated strain, containing or lacking pSfrGR64a::*gfp* were imaged on a Typhoon FLA. Images were processed with Fiji (min intensity, 500, max, 50,000). All the images were treated with the same parameters and taken at the same emission. **(C)** Fluorescence levels (expressed as area under the curve, AUC) for different species carrying pSfr64a::*gfpmut3*. For AUC calculation, fluorescence levels were determined along the growth cycle (24 h) and normalized according to growth absorbance. Results were analyzed statistically by Kruskal-Wallis test, followed by pairwise Wilcoxon rank-sum tests (^***^, *p* < 0.001; ^****^, *p* < 0.0001).

### 3.3 Acquisition of pSfr64a lead to a marked reduction in fitness in *A. tumefaciens*, but not in *R. etli*

Since introduction of pSfr64a has species-specific consequences on conjugative transfer and expression of a foreign gene, we decided to evaluate if it affects differentially the fitness of the host. To do that, we evaluated the maximum specific growth rate (*μ*_max_) in rich medium of *A. tumefaciens* and *R. etli*, either possessing or lacking pSfr64a. As shown in Figure 2A, introduction of pSfr64a has negligible effects on the *μ*_max_ of *R. etli* (from 0.195 to 0.213) but provokes a significant reduction for *A. tumefaciens* (mean *μ*_max_ changes from 0.394 to 0.254 upon acquisition of pSfr64a), when grown in rich medium. A similar situation was also seen when *μ*_max_ was evaluated in minimal medium (Supplementary Figure S1), where acquisition of pSfr64a in *R. etli* provokes weak changes in mean *μ*_max_ (from 0.175 to 0.148), but a highly significant difference for *A. tumefaciens* (from 0.217 to 0.138).

**Figure 2:**
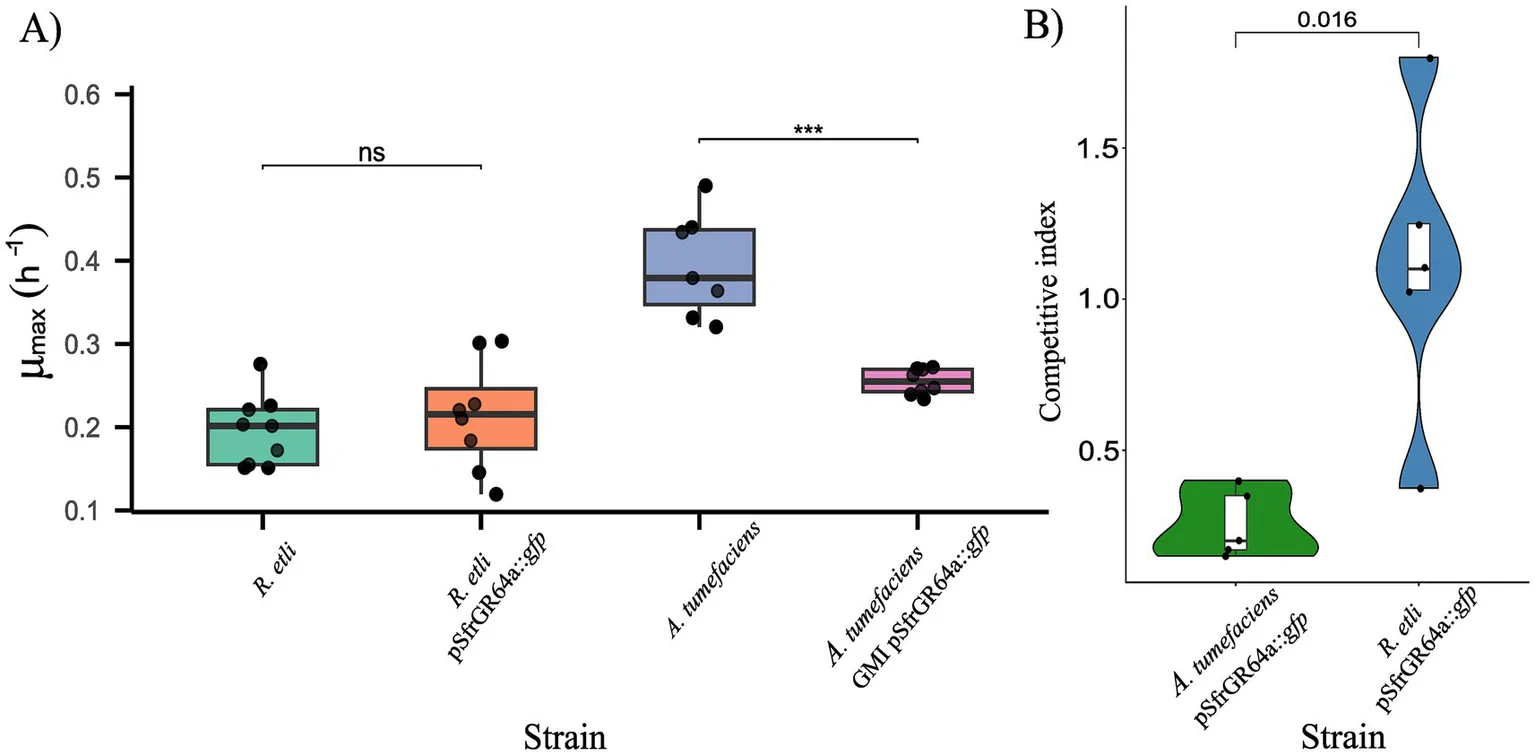
Plasmid pSfr64a has species-specific effects on cell fitness. **(A)** Maximum specific growth rate (*μ*_max_) in rich medium (PY) between different strains. Growth kinetics obtained from at least seven biological replicates, with three technical replicates per each biological replicate. Significant differences were identified by the Wilcoxon test. NS, not significant; ^***^, *p* < 0.0001. **(B)** Determination of competitive index (CI) of strains harboring pSfr64a compared to an isogenic, plasmid-free strain.

Competition assays, where two strains are grown together, allows a direct evaluation of effects on cell fitness. To do that, competition assays in liquid rich medium were set up for combinations *R. etli* CFN2001 vs. *R. etli* CFN2001/pSfr64a and *A. tumefaciens* GMI9023 vs. *A. tumefaciens* GMI9023/pSfr64a (Materials and Methods). Competitive Index (CI) results were obtained after 8 h of growth (Figure 2B). In these experiments, CI > 1 indicate that the plasmid-bearing strain has a fitness advantage over the plasmid-free strain, CI = 1 indicates no fitness difference, while CI < 1 reveals a disadvantage of the plasmid-bearing strain over the plasmid-free strain. As shown in Figure 2B, introduction of pSfr64a in *R. etli* has no effect on competitive ability (mean CI = 1.1 ± 0.5), but its introduction in *A. tumefaciens* has a severe effect on competitive ability (mean CI = 0.25 ± 0.1). It is worth noting that competitive index determinations for *R. etli*, although centered in 1.1 showed a variable behavior, with outliers.

The reduced competitive ability of *A. tumefaciens* GMI9023/pSfr64a may be due to the reduced *μ*_max_ observed in comparison to its plasmid-free derivative. If that were the case, further reductions in CI would be seen if competition experiments were carried out for longer growth periods. When determinations of CI were carried out after 48 and 72 h growth (Materials and Methods) no significant effects in competitive ability of *R. etli* CFN2001/pSfr64a were seen over its plasmid-free derivative (48 h, mean CI = 1.9 ± 0.9; 72 h, mean CI = 1.2 ± 0.05). In contrast, negative effects on competitive ability were seen for *A. tumefaciens* GMI9023/pSfr64a when compared to its plasmid-free derivative (48 h, mean CI = 0.09 ± 0.06; 72 h, mean CI = 0.04 ± 0.04).

### 3.4 Transcriptional landscape of pSfr64a on different hosts

To explore the influence of different chromosomal backgrounds on the transcriptional activity of pSfr64a, we undertook a RNAseq analysis of strains *S. fredii* GR64, *R. etli* CFN2001/pSfr64a, and *A. tumefaciens* GMI9023/pSfr64a, grown to mid-exponential phase in rich medium (Materials and Methods). Reads from each strain corresponding to pSfr64a were extracted, normalized and depicted in Figure 3.

**Figure 3:**
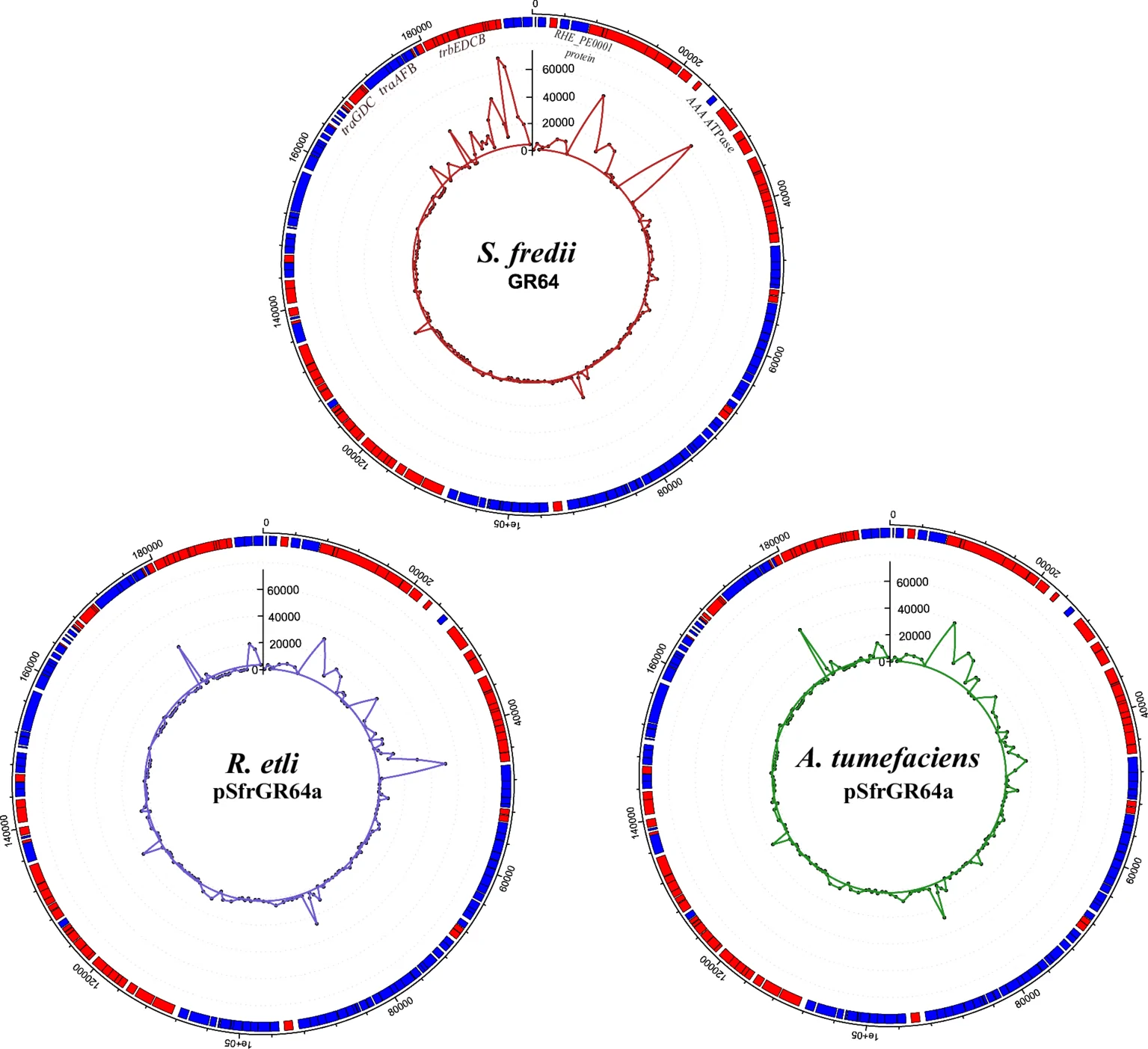
Transcriptional landscape of pSfr64a in different species. Outer circles are a representation of genes on the plasmid (scale in nt). The inner polygonal line in each block represents the transcriptional activity in the corresponding host, expressed as normalized reads (scale, 1–60,000 reads).

In its original host (*S. fredii* GR64), most of the transcriptional activity of pSfr64a is centered in four zones. The largest and most active sector spans from 168 to 189 kb, corresponding to the Dtr (*traCDG* and *traAFBH*) and Mpf (*trbCDEJKLFHI*) systems, relevant for conjugation. The second sector (centered at kb 5), has a weaker activity and contains two genes (a SAM class I methyltransferase and a RHE_PE00001 paralogue); this is the sector where the GFP marker was inserted. The third sector (kb 13 to kb 19) contains genes related to persistence and stability of the plasmid (a DNA methyl cytosine transferase of unknown specificity, a toxin-antitoxin system of the family AbiEii/AbiGii and an ATPase of the megafamily AAA), while the fourth sector (kb 23–25) encompass genes related to IS21.

An analysis of transcriptional levels of pSfr64a when residing in *R. etli* or in *A. tumefaciens* reveals that these sectors are active in both species, but that expression levels are quelled in these hosts. Judging by the number of normalized reads, quelling of transcriptional activity is more severe in *R. etli* than in *A. tumefaciens*.

To validate and extend these findings, a differential expression analysis of pSfr64a in both species was carried out (Materials and Methods), using the basal expression of the genes in *S. fredii* GR64 as reference. Criteria for identification of Differentially Expressed Genes (DEG) used the Benjamini-Hochberg false discovery rate (FDR) correction (Benjamini and Hochberg, 1995), adjusted *p*-value cut off <0.05 and a log_2_fold change (LFC) of 1.5. Results are shown in Figure 4 and in Supplementary Table S4.

**Figure 4:**
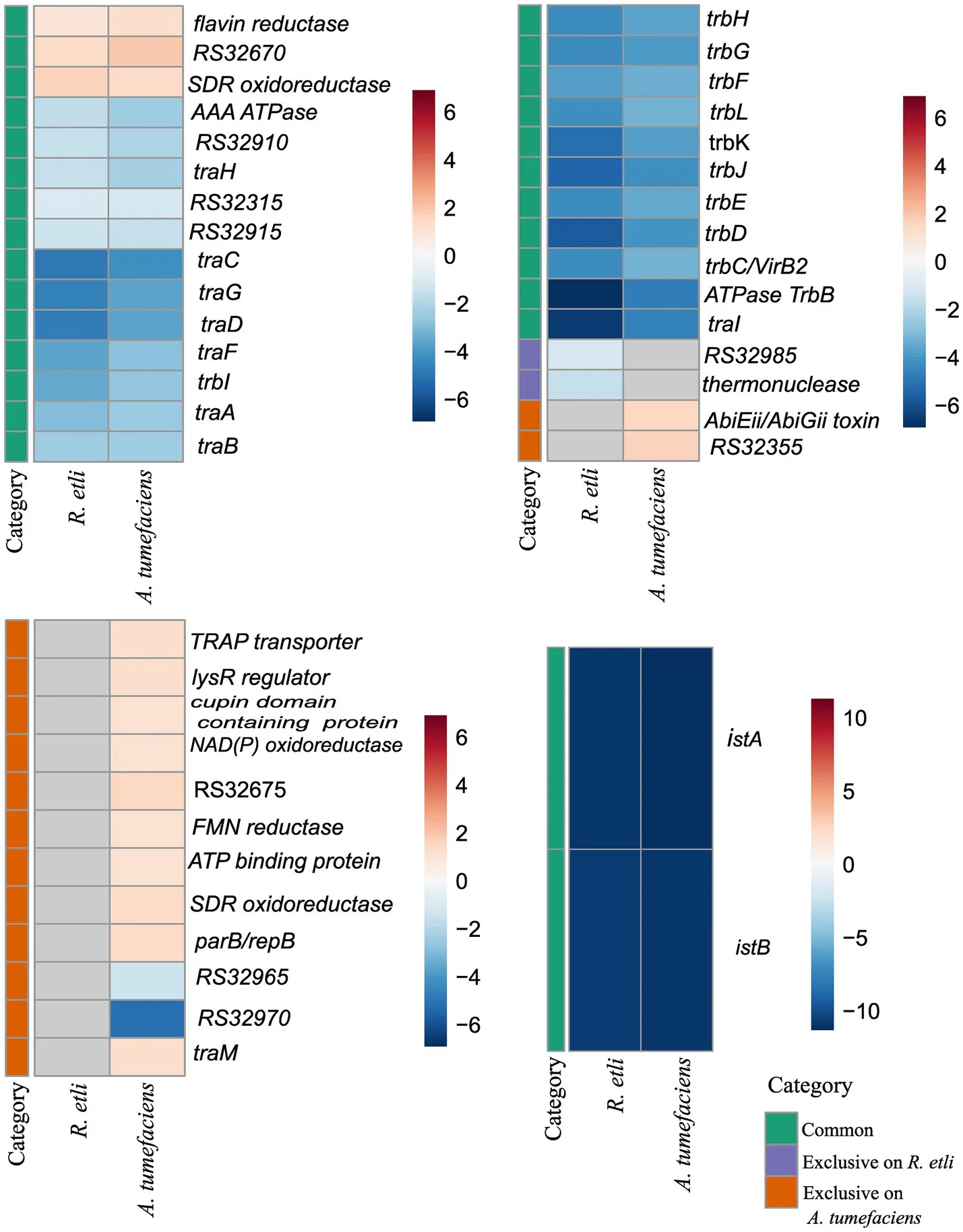
Differential expression analysis of pSfr64a in different hosts. In the heatmaps, DEGs are presented according to the host species, with changes in transcriptional activity expressed as log_2_FoldChange. Red, upregulated; blue, downregulated. Colored vertical bars at the left of each heatmap indicate DEGs common to both species (green), DEGs exclusive for *R. etli* (purple) or exclusive for *A. tumefaciens* (red).

Considering data for both species, a total of 44 pSfr64a genes were classified as DEGs. Of these, 25 were downregulated in both *R. etli* and *A. tumefaciens*, while two were downregulated only in *R. etli* and two in *A. tumefaciens*. All these downregulated DEGs are in the four main sectors of transcriptional activity mentioned above. Twelve genes showed upregulation only in *A. tumefaciens*, while three were upregulated in both species. Surprisingly, the two most downregulated DEGs in both species are *istAB*, related to IS21. Clearly, most of the downregulated DEGs (19 out of 25) are in the conjugative region (*tra* and *trb* genes) of pSfr64a, with a few located in the rest of the plasmid. Upregulated DEGs, observed mainly in *A. tumefaciens*, are located outside of the conjugative region.

A closer look of the conjugative region reveals a general downregulation for these genes, with stronger reductions for *R. etli* (log_2_ fold change range −1.5 to −6.9, corresponding to 2.8–119.4-fold) than for *A. tumefaciens*, which displays a more modest reduction (log_2_ fold change range −2.2 to −4.7, or 4.5–25.9-fold). Among the genes whose transcriptional activity is quelled more strongly in *R. etli* than in *A. tumefaciens*, are Dtr (*traCGD*) and Mpf components (*trbBDJK*), as well as *traI*, that participates in production of AHL. Interestingly, genes regulating the transcription of the conjugative region (*traM* and *traR*) showed only minor variations in expression, suggesting that the reduced expression in the conjugative zone is not due to the absence of these regulatory elements. These data are consistent with the idea of a species-dependent silencing of pSfr64a, particularly for the conjugative region.

### 3.5 Acquisition of pSfrGR64a affects differentially the expression of the host genome

The design of the RNAseq analyses included the obtention of transcriptome data for each complete host genome in the absence of pSfr64a (Materials and Methods). In these analyses, criteria for identification of DEGs used the Benjamini-Hochberg FDR correction, adjusted *p*-value cut off < 0.05 and a log_2_fold change (LFC) of 1.5. For *R. etli*, acquisition of pSfr64a has a modest effect on the expression of the host genome (Figure 5A; Supplementary Table S5). Only 12 DEGs were detected (one downregulated, 11 upregulated), all in plasmids of *R. etli* (the downregulated DEG in pRet42e, the upregulated DEGs in pRet42f). None of these genes are in zones that affect strain fitness in rich medium (Brom et al., 2000; Landeta et al., 2011); these results show that the introduction of pSfr64a does not affect the transcription of genes related with the growth of the strain. As expected from the absence of effect on cell fitness of acquisition of pSfr64a in *R. etli*, no chromosomal DEGs were detected.

**Figure 5:**
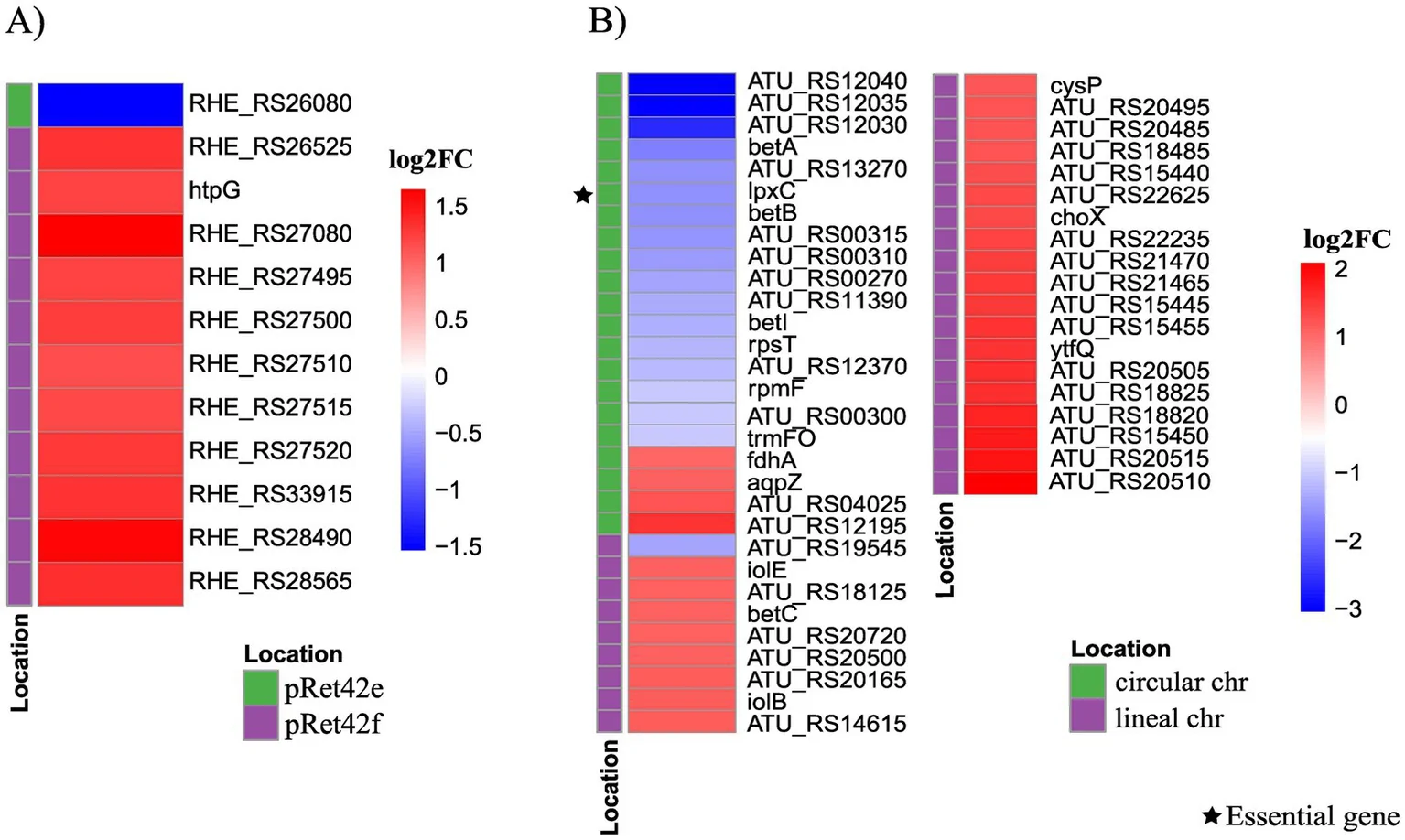
Introduction of pSfr64a has differential effects on the expression of the host genome. For both panels, DEGs are presented in the heatmaps with changes in transcriptional activity expressed as *log2FoldChange*. Red, upregulated; blue, downregulated. **(A)** Effects on *R. etli*. Colored vertical bars at the left of the heatmap indicate location of DEGs (green, pRet42e; purple, pRet42f). **(B)** Effects on *A. tumefaciens*. Colored vertical bars, green circular chromosome; purple, linear chromosome.

In contrast, introduction of pSfr64a in *A. tumefaciens* has drastic effects on the expression of chromosomal genes (Figure 5B; Supplementary Table S6). Overall, 49 chromosomal DEGs were detected (18 downregulated, 31 upregulated). Among the downregulated DEGs, one corresponds to the essential gene *lpxC* (Atu2085), encoding UDP-3-O-acyl-N-acetylglucosamine deacetylase, the first committed step of the lipid A biosynthetic pathway. Downregulation was also observed for ATU_RS19545 (Atu4182) and ATU_RS00270 (Atu0055), both of which encode for nonessential tRNA-Ala TGC, as well as for *rpsT* (Atu0323, encoding the nonessential 30S ribosomal protein S20) and *rpmF* (Atu0112, encoding the nonessential 50S ribosomal protein L32). Reduced expression of *lpxC*, *rpsT* and *rpmF* was also corroborated by RT-qPCR (Supplementary Figure S2). It is plausible that the cumulative effects of downregulation in these genes may be responsible for the negative effects on fitness seen in *A. tumefaciens.*

An unexpected observation stemming from this analysis is a marked regionalization of transcriptional effects. For the downregulated DEGs, 17 out of 18 are located on the *A. tumefaciens* circular chromosome, while 27 out of 31 upregulated DEGs reside on the linear chromosome. Possible reasons for this will be presented in the Discussion.

### 3.6 MucR participates in silencing of pSfr64a, but not for the conjugative zone

Having observed a species-specific quelling of transcriptional activity of pSfr64a, an attempt was made to determine the system responsible for this phenomenon. The only recognized xenogeneic silencer in Rhizobiales is MucR (known as RosR in *R. etli*). To evaluate the possible role of MucR in pSfr64a silencing, a knockout mutant of *mucR/rosR* was generated by vector-insertion mutagenesis (Materials and Methods) in *R. etli* CFN2001/pSfr64a::*gfp*. The mutant showed phenotypes typical of a *rosR* mutant in *R. etli* (dome-shaped colonies and hydrophobic cells prone to flocculate in liquid cultures, Araujo et al., 1994; Bittinger and Handelsman, 2000; Bittinger et al., 1997). To assess the effect of a *mucR* mutation, determinations of conjugation frequency, expression of GFP and RT-qPCR analysis of previously studied plasmid genes were performed.

For the conjugation assays, we decided, based on the observation reported here that *traI*, a gene that participates in production of AHL is strongly repressed, to include a condition that replenishes AHL, namely addition of *S. fredii* spent culture medium. Addition of *S. fredii* spent culture medium to *R. etli* CFN2001/pSfr64a::*gfp* increase plasmid transfer one hundred-fold (Table 2, lines 3 and 4), indicating that reduced AHL levels is one of the factors that limit pSfr64a conjugative transfer in *R. etli*.

**Table 2:** Conjugation efficiency of pSfr64a is not restored to normal levels in a *R. etli* mucR mutant.

Donor	Spent culture medium from *S. fredii*	Conjugation frequency (×10^−4^ ± SD)^a^
*S. fredii* GR64-3	−	283 ± 280
*S. fredii* GR64-3	+	1,100 ± 900
*R. etli* CFN2001/pSfr64a::*gfp*	−	0.0003 ± 0.0003
*R. etli* CFN2001/pSfr64a::*gfp*	+	0.08 ± 0.01
*R. etli* CFN2001 *mucR*/pSfr64a::*gfp*	−	0.01 ± 0.01
*R. etli* CFN2001 *mucR*/pSfr64a::*gfp*	+	0.7 ± 0.7

^a^Conjugation frequency is expressed as transconjugants per recipient cell. Data are means of four biological replicates ± standard deviation. All matings were done with *A. tumefaciens* UIA143 as recipient; transferred plasmid was pSfr64a::*gfp*.

Regarding the effect of a mutation in *mucR*, this modification also increases transfer of pSfr64a one hundred-fold (Table 2, lines 3 and 5). Combining the effects of addition of spent culture medium with a mutation in *mucR* has a nonadditive effect, increasing conjugation of pSfr64a one thousand-fold (Table 2, lines 3 and 6). Since conjugation is not restored to normal levels, this indicates that other factors, besides AHL production and silencing by MucR are still limiting conjugative transfer in this host.

Regarding expression of *gfp*, measurements of fluorescence levels revealed a significant increase in fluorescence for *R. etli* CFN2001 *mucR*/pSfr64a::*gfp* compared to *R. etli* CFN2001/pSfr64a::*gfp*, indicating that absence of MucR relieves silencing of the GFP reporter gene (Figure 6).

**Figure 6:**
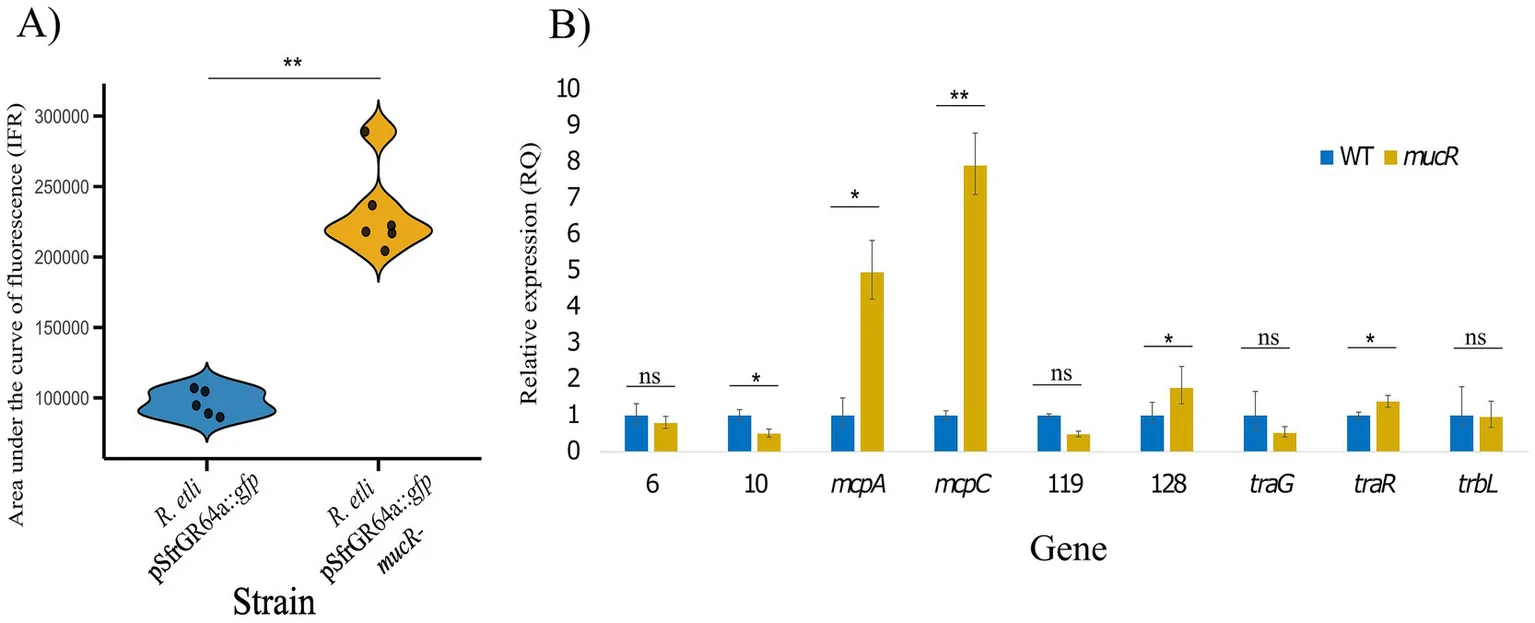
**(A)** A mutation in *mucR* partially relieves silencing in the expression of GFP for *R. etli* CFN2001. CFN2001-4, *R. etli* CFN2001/pSfr64a::*gfp*; CFN2001-7, *R. etli* CFN2001 *mucR*/pSfr64a::*gfp.* Values correspond to three biological replicates and are expressed as normalized area under the curve (AUC) of fluorescence observed (IFR/DO600), for a complete growth cycle (24 h). Differences were highly significant by a Kruskal-Wallis test (^**^, *p* < 0.01). **(B)** Effect of a mutation in *mucR* on the expression levels for nine different regions in pSfr64a. Expression levels were evaluated by RT-qPCR, using three biological replicates (with three technical replicates) for each gene. Expression levels for each gene were normalized using the values for the *rpoA* gene, values for *R. etli* CFN2001/pSfr64a::*gfp* (wt) were set to one of RQ and then compared with the expression levels of *R. etli* CFN2001*mucR*/pSfr64a::*gfp* (*mucR*). Average levels of expression (+/− SD) are shown. Significant differences were identified by pairwise Student *t*-tests. NS, not significant, ^*^, *p* < 0.05, ^**^, *p* < 0.01.

Given our previous findings, no major changes were expected for the nine genes tested on pSfr64a::*gfp* upon mutation of *mucR.* Unexpectedly, two genes with high AT content (44% in *mcpA* and 41% *mcpC*), relative to CFN2001 average AT content, showed 4–7-fold increases in expression compared to the parental strain (Figure 5B). These genes (*mcpA* and *mcpC*), encode methyl-accepting chemotaxis proteins (Salah Ud-Din and Roujeinikova, 2017). Our findings are consistent with a report about MucR control of the expression of *mcp* genes in *R. leguminosarum* (Rachwał et al., 2015).

We conclude that MucR functions as a xenogeneic silencer in the *R. etli* CFN2001. However, it is not responsible for the widespread transcriptional silencing observed. Possible alternative systems will be presented in the Discussion.

## 4 Discussion

In this work, we show that the consequences of transfer of a large rhizobial plasmid is strongly dependent on the host that functions as a recipient. When pSfr64a was transferred to *R. etli*, the plasmid experiences a severe reduction in conjugative ability, to nearly undetectable levels, but when the plasmid enters *A. tumefaciens*, pSfr64a retains a very high conjugative ability. A possible explanation for this phenomenon might be that there are other elements, not encoded in pSfr64a, that influence its conjugative ability, and that these elements are located elsewhere in the genomes of both *S. fredii* GR64 and *A. tumefaciens*, but absent in *R. etli.* Four arguments militate against such interpretation. The first argument, obtained in previous work (Cervantes et al., 2019), is that when three other possible regulators in *S. fredii* that might influence conjugative ability were introduced in *R. etli* harboring pSfr64a, the plasmid still displayed a very low conjugative ability. Two other arguments, obtained in this work, are related to the extent and characteristics of regions that experience reduced transcriptional activity. If it were a phenomenon affecting only conjugative ability, it should not affect other genes unrelated to this function. However, upon introduction of a transcriptionally autonomous marker gene (*gfpmut3*) into pSfr64a, a marked reduction in fluorescence was seen in *R. etli*, but not in either *S. fredii* or in *A. tumefaciens*. Transcriptional analysis of pSfr64a in its original host, reveals that transcription is concentrated in four sectors; one of these corresponds to the conjugative region, but the other sectors were unrelated to conjugation (uncertain status, persistence and maintenance of the plasmid, and transposition). All these sectors display a severe reduction in expression when pSfr64a resides in *R. etli*, but a more modest reduction when plasmid expression was analyzed in *A. tumefaciens*. Finally, the reduced conjugative ability of pSfr64a in *R. etli* is partially relieved by restoring the presence of AHL (addition of *S. fredii* spent culture medium), but also by a mutation in *mucR*, the sole xenogeneic silencer identified thus far in rhizobiales. We think that these observations are better explained by postulating a global quelling of the major transcriptional sectors in pSfr64a, operating a very high efficiency in *R. etli* but at a lower extent in *A. tumefaciens.*

Under this view, the marked reduction in the expression of the conjugative sectors in *R. etli*, particularly for Dtr and Mpf components, as well as for *traI*, explain convincingly the absence of high-frequency transfer of pSfr64a. In *A. tumefaciens*, expression of these genes is reduced, but at a more modest extent, retaining high-frequency transfer. The ability of pSfr64a to transfer from *A. tumefaciens*, even with the reduced level expression of the conjugative genes, suggest permissive regulatory thresholds, where even low transcriptional levels suffice for transfer. This observation is consistent with previous findings in *A. tumefaciens* conjugative systems, where even modest expression levels of *tra* genes can still support DNA transfer (Mhedbi-Hajri et al., 2016). Also, in Ti plasmids systems, conjugative transfer is regulated via AHL-TraR QS circuit, but basal levels of *traR* can still activate transfer in the absence of full induction (Su et al., 2008).

The nature of the system that quells transcriptional activity of pSfr64a in *R. etli* is still unknown. Studies of other plasmids, generally of small size in enterobacteria, have focused on the participation of xenogeneic silencers such as H-NS (Dorman, 2007; Dorman and Kane, 2009; Dorman, 2014; Lucchini et al., 2006; Navarre et al., 2006; Navarre, 2016). However, genes encoding proteins such as H-NS or HU/IHF are absent from rhizobial genomes (Perez-Rueda and Ibarra, 2015). Thus far, the sole xenogeneic silencer identified in rhizobial genomes or in the related genus Brucella is MucR (Baglivo et al., 2024; Baglivo et al., 2017; Jiao and Tian, 2020; Pirone et al., 2018). MucR may play a role in the phenomenon described here, but it is not the sole factor. Although a mutation in *mucR* of *R. etli* restores GFP production, it has a more modest effect in restoring conjugative ability. In enterobacteria, it has been described that genome methylation may have a role in transcriptional silencing (Casadesús and Sánchez-Romero, 2022; Gao et al., 2023). For rhizobiales, the study of DNA methylation is still scarce, although recent studies (Afonin et al., 2021; DiCenzo et al., 2022; Passeri et al., 2025) have identified genome methylation in species of this group, generating mainly N^4^-methylcytosine and N^6^-methyladenine modifications, and the conspicuous absence of 5-methylcytosine. Preliminary experiments reveal that addition of 5-azacytidine (an inhibitor of 5-methylcytosine) to *R. etli* CFN2001/pSfr64a do not affect neither growth nor expression of conjugative transfer genes (data not shown). Experiments are underway to evaluate the location and extent of epigenome modifications in the strains described here.

What happen if quelling of plasmid gene expression is not efficient? Data for many plasmid systems has shown that high level expression may be burdensome for the host cell, particularly for genes participating in conjugation (Curtsinger et al., 2024). In this regard, in *R. etli* (where quelling occurs efficiently) no effects of pSfr64a carriage were detected, neither for growth rate nor for competitive ability. Consistent with this, effects of pSfr64a on *R. etli* host genome were relatively modest: no chromosomal gene was affected, only a few genes in the other plasmids of *R. etli* displayed differential expression. In contrast, in *A. tumefaciens* (where quelling occurs less efficiently), carriage of pSfr64a entails significant effects on fitness, reducing host growth rate and competitive ability. Moreover, altered expression of genes in the host genome is more severe than in *R. etli*, reducing the expression of chromosomal genes that include an essential gene (*lpxC*) and other genes (Atu4182, Atu0055, *rpsT* and *rpmF*) that accumulatively may affect fitness.

Consequently, the profound impact on the host transcriptome, specifically in *A. tumefaciens*, reveals that the presence of the megaplasmid induces a global stress response that extends to both the linear and the circular chromosomes (Figure 5). This suggests that the failure of the silencing mechanism leads to a loss of homeostasis, forcing the host to redirect resources to manage the exogenous DNA load (Navarre, 2016; Doyle et al., 2007). Unlike *R. etli*, where silencing is fine-tuned to allow coexistence without drastic fitness loss, the transcriptional reorganization observed in Agrobacterium highlights the biological cost of an uncontrolled “selfish mode” of the plasmid (Rachwał et al., 2015; San Millan and MacLean, 2017).

Fitness effects of rhizobial megaplasmids have been an understudied topic. Besides the example reported here, there are only two precedents. When pSymB of *Sinorhizobium meliloti* was introduced into *A. tumefaciens*, a negative effect on fitness was detected (Finan et al., 1986). Carriage of pTi of *A. tumefaciens* entails a significant cost, detectable in resource-limiting conditions; this cost is augmented upon induction of genes participating in conjugation (Platt et al., 2012).

An intriguing observation made during these studies was that, when pSfr64a was introduced into *A. tumefaciens*, there is a regionalization of transcriptional effects on the host genome. Virtually all the downregulated DEGs are located on the circular chromosome, while the upregulated DEGs are in the linear chromosome. Although the reasons for this remain obscure, at least two possible reasons can be envisioned. One may be related to the differing topology between the two chromosomes, where linearity may facilitate upregulation. Another alternative may be the distinct evolutionary origin of these replicons. The circular chromosome appears to be the original chromosome, based on DnaA dependence for its replication, while the linear chromosome has a more plasmid-like origin, based on its reliance on a RepABC system, typical for megaplasmids (diCenzo and Finan, 2017). It might be that regionalization is a consequence of these evolutionary origins, where plasmid acquisition tends to have a negative effect on chromosomes while positive, or even cooperative effects are common between plasmids. It would be interesting to evaluate what changes, if any, occur in the DEGs generated by pSfr64a in an *A. tumefaciens* derivative in which the circular and linear chromosomes are fused in a single, linear chromosome (Liao et al., 2022).

Integrating these observations on structural regionalization and fitness costs, the acquisition of a megaplasmid represents a fundamental evolutionary conflict of interest. A plasmid with high expression of genes can assure its own transfer and existence even if it causes a high metabolic cost in the host, acting in a ‘selfish mode’ when the growth medium is rich (Platt et al., 2012; Su et al., 2008). In return, a host with adapting mechanisms and modulation systems (such as xenogeneic silencing), can control the transfer of the plasmid and reduce the biological cost associated with a megaplasmid of this size (Dorman, 2007; Navarre et al., 2006). This model suggests that xenogeneic silencing does not act uniformly over all the plasmid, but rather over genes whose activation could compromise the homeostasis of the host (Thomas and Nielsen, 2005; Mhedbi-Hajri et al., 2016).

Ultimately, the data presented here demonstrate that the conjugative ability of pSfr64a do not depend only on the Quorum Sensing (QS) system, but it is integrated into a hierarchical, two-tiered regulatory architecture, where xenogeneic silencing (and potentially methylation) represents the upper layer of control (Lucchini et al., 2006; Dorman, 2007). In this framework, QS does not stop being functional, but works in a subordinate way, driving transfer only when the basal silencing is relieved (Perez-Rueda and Ibarra, 2015). This kind of layered regulation has been widely described in genes obtained via HGT, where the host cell imposes a global control to avoid deleterious effects, allowing subsequently the action of specific regulators under permissive conditions (Navarre, 2016; Thomas and Nielsen, 2005). In this sense, the inability of the QS system to work on pSfr64a in the genomic background of *R. etli* reveals a restriction imposed by these regulatory networks, of higher hierarchy (Mhedbi-Hajri et al., 2016). Future work will be devoted to exploring the generality of such systems for rhizobial plasmids.

## Data Availability

The RNAseq data generated in this study are available in the NCBI SRA database under BioProject accession PRJNA1428832. Additional data for the experiments reported here are available upon request to the corresponding author.

## Footnotes

1
https://primer3.org/
