# Supplementary material for: Transcriptional silencing of a conjugative Sinorhizobium megaplasmid upon transfer to different rhizobial species
Source: Front Microbiol. 2026 Oct 22;17:1977405. doi: 10.3389/fmicb.2026.1977405 (PMC13736051; doi:10.3389/fmicb.2026.1977405)
Supplement: Supplementary file 1 [file Data_Sheet_1.PDF]

Supplementary Material

1 Supplementary Figures

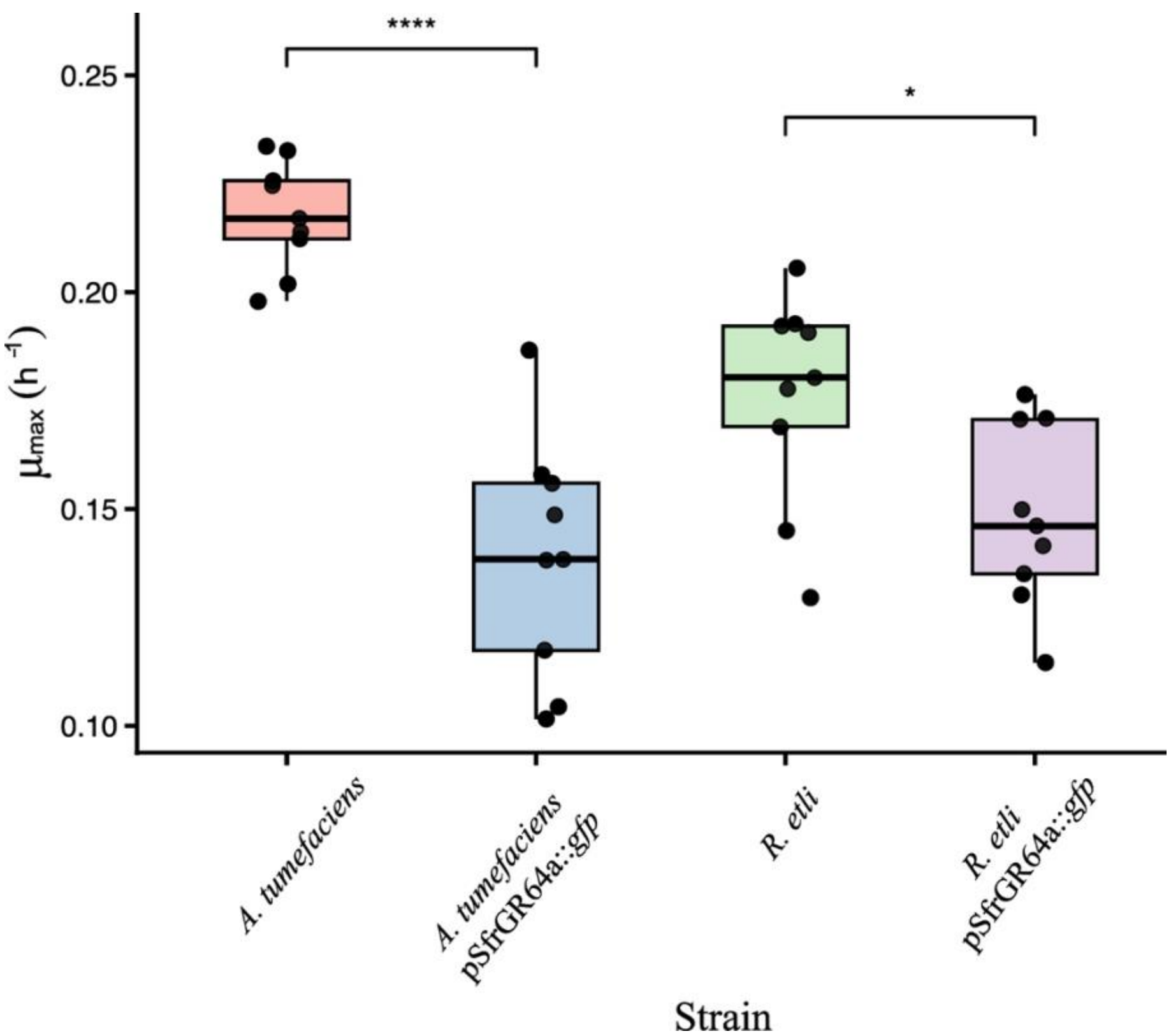

Supplementary Figure 1. Presence of plasmid pSfr64a affects fitness in minimal medium. Maximum specific growth rate (μmax) in minimal medium (MMY) between different strains. Growth kinetics obtained from at least nine biological replicates, with three technical replicates per each biological replicate. Significant differences were identified by the Wilcoxon test. *, $p < 0.05$, ***, $p < 0.0001$.

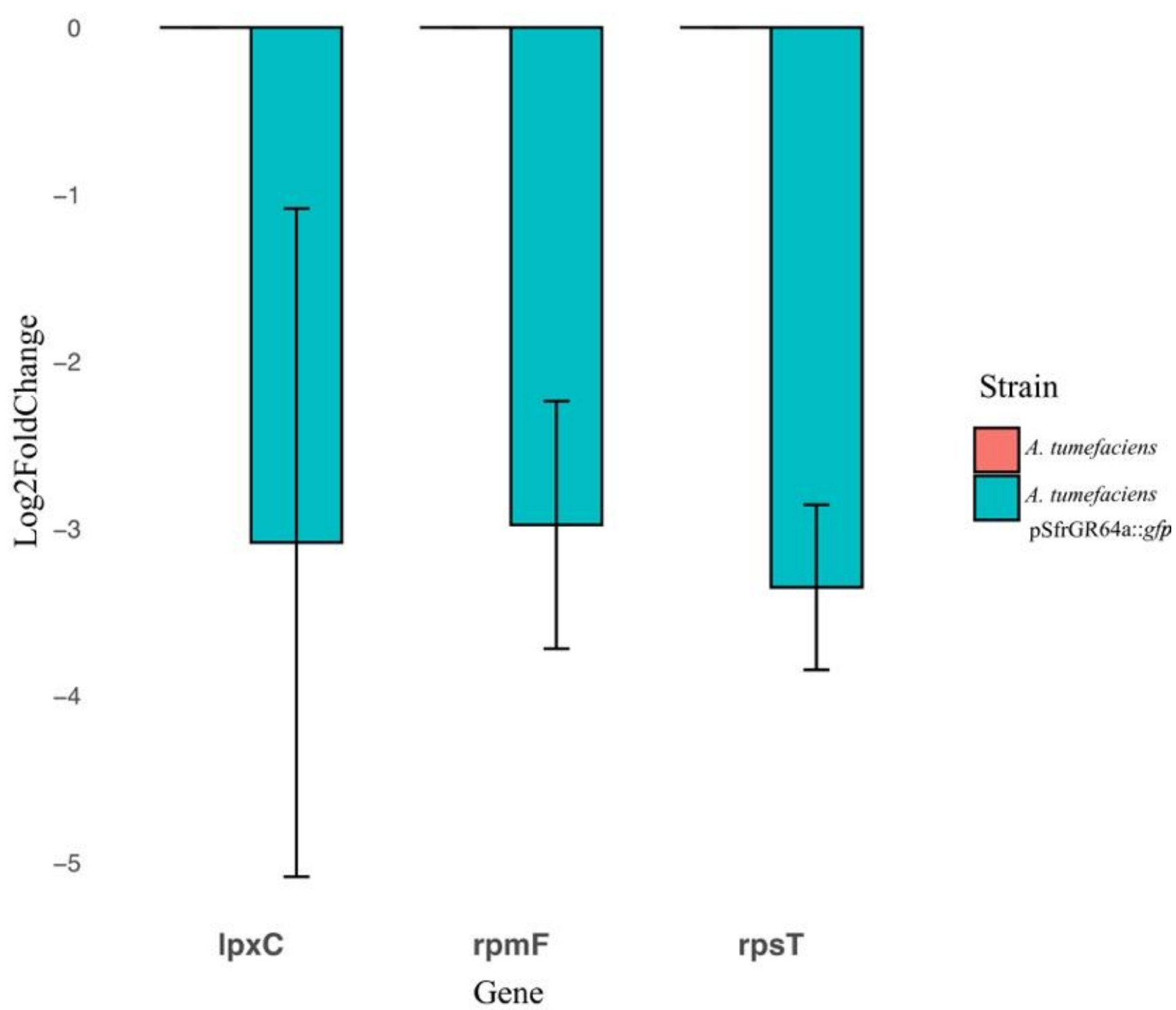

Supplementary Figure 2. Verification of *A. tumefaciens* chromosomal DEGs by RT-qPCR. Expression levels were evaluated by RT-qPCR, using two biological replicates (with three technical replicates) for each gene. Expression levels for each gene were normalized using the values for the *rpoA* gene, values for *A. tumefaciens* GMI9023 were set to zero of log2fold change and then compared with the expression levels of *A. tumefaciens* GMI9023/ pSfr64a*::gfp*. Average log2fold changes (+/- SD) are shown.
